# Children With Disruptive Mood Dysregulation Disorder and Psychopathological Risk in Their Mothers: The Function of Global DNA Methylation

**DOI:** 10.3389/fpsyt.2021.593500

**Published:** 2021-01-27

**Authors:** Valeria Carola, Silvia Cimino, Silvia Bussone, Luca Cerniglia, Renata Tambelli

**Affiliations:** ^1^Department of Dynamic and Clinical Psychology, Sapienza University of Rome, Rome, Italy; ^2^IRCCS Santa Lucia Foundation, Rome, Italy; ^3^Faculty of Psychology, International Telematic University Uninettuno, Rome, Italy

**Keywords:** global DNA methylation, epigenetics, saliva, disruptive mood dysregulation disorder, clinical psychology, SCL-90-R, CBCL/6–18, ECR-RC

## Abstract

Epigenetic mechanisms, in particular DNA methylation, have been implicated in the etiopathogenesis of psychopathologies in adulthood. The significance of this mechanism in child psychopathologies, however, is much less recognized. Here, we examined whether global DNA methylation alteration was associated with the presence of disruptive mood dysregulation disorder (DMDD) in children. Moreover, in light of the relevance of the interplay between children and parents for the onset and maintaining of psychopathology during development, we measured the association between psychological symptoms, attachment styles, and global DNA methylation levels in healthy and DMDD mother-child dyads (mothers: *N* = 126, age = 38.3 ± 2.5 years; children: *N* = 150, age = 8.2 ± 0.9 years, gender ratio [f/m] = 72/78). We did not observe any significant differences in global DNA methylation levels in DMDD children when compared with healthy peers, and children's symptoms did not correlate with variations in this parameter. The mothers showed different levels of psychological symptomatology. Notably, mothers with high psychological symptomatology showed the lowest levels of global DNA methylation. Maternal global DNA methylation levels were associated with maternal hostility, interpersonal sensitivity, psychoticism, and general severity index. Moreover, we found an effect of maternal mental health on the severity of children's symptoms, independently from both maternal and child DNA methylation levels. Despite here DNA methylation does not appear to be involved in the maternal inheritance of vulnerability to depression, this biological link could still arise in later stages of the child's development.

## Introduction

Bio-psycho-social approaches that highlight the interplay of the child, parent, family, and other environmental factors are commonly used to depict the complexity of psychopathology during child development (1, 2). The Developmental Psychopathology clinical and theoretical framework has widely demonstrated that parents and children often share psychopathological risk (3). This fact can be due to shared genetic set predisposing to psychopathology, disrupted parenting that predicts offspring symptoms, shared stressful environments, and lack of social support (4). In line with this standpoint, the Transactional framework (5, 6) highlighted how children's emotional and behavioral characteristics and parent's ability to act as a secure base can be considered as mutually influencing, and that parental and offspring psychopathology appear to reciprocally reinforce (7).

Despite the copious work on childhood psychopathology and its long-term consequences, little is known about the neurobiological mechanisms that have been implicated in and modulate the psychological symptoms in childhood, and through which the environmental familial interactions affect the developmental biological trajectories in children. Among the different epigenetic mechanisms, such as histone modifications (e.g., histone acetylation, methylation, ubiquitylation, phosphorylation, and sumoylation) and DNA methylation, DNA methylation has been strongly implicated in the pathogenesis of a variety of psychiatric disorders (8, 9) and, more importantly, in the “translation” of early-life environmental (e.g., stress) events into biological and psychological alterations during adulthood (10, 11). DNA methylation is a repressive epigenetic marker, entailing the covalent addition of a methyl group to the C-5 position of the cytosine ring in DNA by DNA methyltransferases (12). Increased methylation in these regions is typically associated with the inhibition of gene transcription (e.g., gene silencing) and chromatin compaction (13).

Although few studies have examined the contribution of DNA methylation to childhood psychopathology using a candidate gene approach—i.e., the methylation of a single gene or a pool of genes (7, 14)—none has determined whether global DNA methylation levels vary in clinical samples during childhood. In children, disruptive mood dysregulation disorder (DMDD) is a clinical condition that is characterized by severe impairments in emotional and behavioral regulatory processes (15, 16). In the DSM-5, DMDD is defined as a type of depressive disorder diagnosis for youths, and DMDD children are at risk for developing depression and anxiety in adulthood (17).

In light of recent evidence consistently describing an alteration of DNA methylation in mood disorders in adulthood (18), here we hypothesize that children with a diagnosis of DMDD experience decreased global DNA methylation levels and such changes are negatively associated with psychological symptoms and attachment style.

Considering studies reporting that parents of DMDD children may suffer psychopathological symptoms (19), and studies reporting decreased global DNA methylation levels in presence of psychological alterations in adulthood (18, 20), we hypothesize that mothers of DMDD children show psychopathological alterations associated with decreased global DNA methylation levels.

Further, we assume the existence of an effect of maternal mental health on the child's symptoms. Preclinical and clinical evidence describes DNA methylation as one of the potential mechanisms mediating the enduring effects of early-life experience on health outcomes. Moreover, parents' experiences may transfer epigenetic marks that impact offspring development alone and in interaction with offspring exposure to perinatal and childhood stress (11, 21). Based on these premises we hypothesize that the effect of maternal mental health on child's symptoms may be mediated by maternal and/or childhood DNA methylation.

In this study, we measured this parameter in healthy and clinical (DMDD) mother-child dyads and examined its association with maternal and child psychological symptoms and attachment style.

## Methods

### Participants

Participants included a total of 150 children, 72 females and 78 males, aged from 8 to 9 years (*M* = 8.2; SD = 0.9), and their mothers (*M* = 38.3 years; SD = 2.5). The clinical group was composed of 85 children who were diagnosed for the DMDD without comorbidity by a group of psychologists according to the DSM-5 (22), recruited from the mental health services of Central Italy. The subjects did not report history of suicidal behavior and were not pursuing any pharmacological treatment. The healthy control group included 65 children who reported no psychopathological symptoms, recruited from schools of Central Italy. The families were 100% Caucasian, and most of them had a middle-high socioeconomic level according to the Hollingshead's social status index (25,000–30,000 Euros per year) and educational level (high school or university) (23). In the majority (96%) of families, no divorce/separation was detected. Furthermore, 86.7% of children were first-born for both parents. Confounding variables (such as alcohol use, smoking, drugs of abuse, current medical illness, traumatic experiences, and social-economic status) were assessed in mothers through *ad-hoc* anamnestic questionnaire specifically created for this study. Mothers read and signed the informed consent before test administration. Once the parents signed the informed consent, an oral explanation of the project was given to children. All questionnaires listed below were filled at home. On a different day, epithelial cell samples were obtained through buccal swabs from mothers and children. In accordance with the Declaration of Helsinki, this study was approved by the Ethical Committee of the Department of Dynamic and Clinical Psychology at Sapienza, University of Rome (27/2016).

### Clinical Assessment

#### Emotional and Behavioral Functioning, and Attachment Style in Children

Mothers (*N* = 126) filled out the Italian version of the Child Behavior Check-List/6–18 [CBCL/6-8, (24); Italian version, (25)], that is one of the most used instrument to measure childhood and adolescent psychopathology in both clinical and normative samples. The CBCL/6–18 is a 113-item informant-report questionnaire that requires parents (mothers and fathers independently) to rate specific emotional/behavioral problems of their child during the past 6 months. Items are rated on a three-point Likert scale ranging from 0 (not true) to 2 (very true or often true), and are clustered into eight syndrome scales: anxious/depressed, withdrawn/depressed, somatic complaints, social problems, thought problems, attention problems, rule-breaking behavior, and aggressive behavior. In this questionnaire, anxious/depressed, withdrawn/depressed, and somatic complaints scales are grouped into the subscale of internalizing problems, whereas rule-breaking behavior and aggressive behavior scale are grouped into the subscale of externalizing problems. Social problems, thought problems, and attention problems (not grouped into any subscale) are also evaluated by this survey. DSM-5 oriented scales (depressive problems, anxiety problems, somatic problems, attention deficit/hyperactivity problems, oppositional defiant problems, and conduct problems) were also used.

Attachment style in children was measured through the Italian version (short form) of the Experiences in Close Relationships-Revised Child questionnaire [ECR-RC, (26); Italian version, (27)]. The self-report short form of ECR-RC is made up of 12 items (six for anxiety and six for avoidance) on a five-point Likert scale. For our purpose we decided to use the ECR-RC categorically, by dividing the four attachment styles in “secure attachment” and, on the other hand “insecure attachment,” which includes fearful, preoccupied and dismissing attachment styles. Findings across studies suggest the ECR-RC is a valuable tool for measuring anxious and avoidant attachment to parents in middle childhood and adolescence. It shows excellent reliability and validity (26, 28), showing a Cronbach's alpha of 0.83 for attachment anxiety and 0.85 for avoidance (26). On the other hand, the Italian validation of the ECR-RC exhibited a Cronbach's alpha of 0.95 for attachment anxiety, 0.87 for avoidance and 0.90 for secure attachment (27).

#### Psychopathological Symptoms in Mothers

Mothers were administered with the Italian version of Symptom Check-List-90 Revised [SCL-90-R, (29); Italian version, (30, 31)]. SCL-90-R is a 90-item self-report that measures psychopathological symptoms and psychological distress in adults from normative and clinical populations. The SCL-90-R is rated on a Likert scale of 0 (not at all) to 4 (extremely), and asks participants to report if they have suffered in the past week from symptoms of somatization (e.g., headaches), obsessive-compulsivity (e.g., having to check and double-check what you do), interpersonal sensitivity (e.g., feeling that people are unfriendly or dislike you), depression (e.g., feeling blue), anxiety scale (e.g., feeling fearful), hostility (e.g., having urges to beat, injure, or harm someone), phobic anxiety (e.g., feeling afraid to go out of your house alone), paranoid ideation (e.g., persecutory beliefs concerning a perceived threat toward oneself), and psychoticism (e.g., having thoughts that are not your own). Aside from these nine primary scales, the questionnaire provides a global severity index (GSI), which is used to determine the symptomatology severity and degree of psychological distress linked to the symptoms. Finally, we divided the mothers into two subgroups based on their GSI score. According to commonly used criteria for interpreting the GSI scores (31), mothers with raw GSI score between 0 and 0.78 (T score < 55) were included in the low GSI group and considered asymptomatic (*N* = 61), whereas mothers with raw score between 0.79 and 1.70 (T score > 55) were included in the high GSI group and considered symptomatic (*N* = 67).

### Procedure for Biological Sampling

Epithelial cell samples from children and mothers were collected by buccal swabs (Isohelix Swab Pack, Cell Product Ltd, Harriestam, UK). All subjects were asked not to eat, drink (except water), and brush their teeth for at least 1 h before sampling. The biological samplings were slightly chilled by normative ice (+4°C) and transported to the laboratories of the co-author (V.C.) for further processing. After buccal swabs were gathered, mothers and children independently filled out self-report and report form questionnaires (described above).

#### DNA Purification and Dot Blot Assay

Buccal cell DNA isolation was performed using the Buccal-Prep Plus DNA isolation kit according to the manufacturer's instructions. DNA yield and quality were determined by Nanodrop absorbance at 260 and 280 nm. The yield of DNA was between 3 and 10 μg, and we did not find significant differences in yield between mothers and children samples. DNA samples with ratio of absorbance A260/A280 within the range of 1.7–2.0 were considered of good quality and included in the analysis. Dot blot analysis was performed to detect DNA methylation by 5-methylcytosines levels. The protocol was adapted from the one of Cui et al. (32). Each sample was denatured for 10 min at 99°C and then neutralized for 10 min at 4°C. After that, 1 μl of diluted genomic DNA was spotted in duplicates on an N+ nitrocellulose membrane (Roche, Italy), immobilized by UV cross-link (5000 microJoules/cm2 at 254 nm) and incubated after blocking with anti-5-methylcytosine (Diagenode, Belgium). In parallel, a standard curve was spotted with increasing concentrations of DNA sample at known methylation level (Sigma-Aldrich, Germany). Dots were detected with the aid of iBRIGHT (ThermoFisher, USA), using ECL-based reagents (ThermoFisher, USA) with appropriate secondary antibody, HRP (horseradish peroxidase) conjugated. Optical density was measured with Image J software (NIH, Maryland, USA) and normalized for the individual DNA quantity loaded based on their original concentration). Levels of methylation were deducted by interpolation with the standard curve. For this parameter, if a value was three standard deviations away from the mean of the group that data point was identified as an outlier and excluded from the analysis (1 child and 3 mothers).

### Statistical Analysis

The normal distribution of the measured parameters was assessed by Kolmogorov-Smirnov test.

In light of the evidence that global DNA methylation in the blood differs between clinical syndromes (e.g., major depression) and that it mediates the effects of primary affective relationships (e.g., attachment) on the child development (33), we evaluated if this parameter was differentially modulated in distinct samples of children characterized by different diagnosis (healthy vs. clinical-DMDD) and attachment style (secure vs. insecure). Main and interaction effects of attachment style and child clinical symptoms on child global DNA methylation levels were calculated by using two-way analysis of variance (ANOVA). In case of significant main and/or interaction effect, *post-hoc* comparisons were performed by Duncan's test. To evaluate whether the psychological status of the children (CBCL/6-8 subscales scores) was associated with global DNA methylation levels, bivariate correlations (Pearson r) analyses were performed.

To evaluate the modulation of the maternal symptomatology and the attachment style on maternal global DNA methylation level, this parameter was assessed in mothers characterized by different levels of distress due to symptoms severity (high vs. low GSI) and attachment style (secure vs. insecure) by two-way ANOVA. In case of significant main and/or interaction effect, *post-hoc* comparisons were performed by Duncan's test. Further, to evaluate whether the psychological status of the mothers (SCL-90-R) was associated with global DNA methylation levels, bivariate correlations (Pearson r) were performed.

Multiple regression analyses, followed by mediation analysis (34), were used to estimate the contribution of maternal distress due to symptoms severity (GSI scores) to child symptomatology (CBCL/6-18 subscales scores), over and above the contribution of maternal and child global DNA methylation. Finally, to evaluate the association between maternal and child global DNA methylation levels, bivariate correlation (Pearson r) was performed.

Statistical analyses were carried out with the help of Statistica software Version 12.0 (StatSoft, Tulsa, OK, USA) and SPSS for Windows, version 25.0.

## Results

### Global DNA Methylation in Children and Its Association With Psychopathological Symptoms

**Figure 1:**
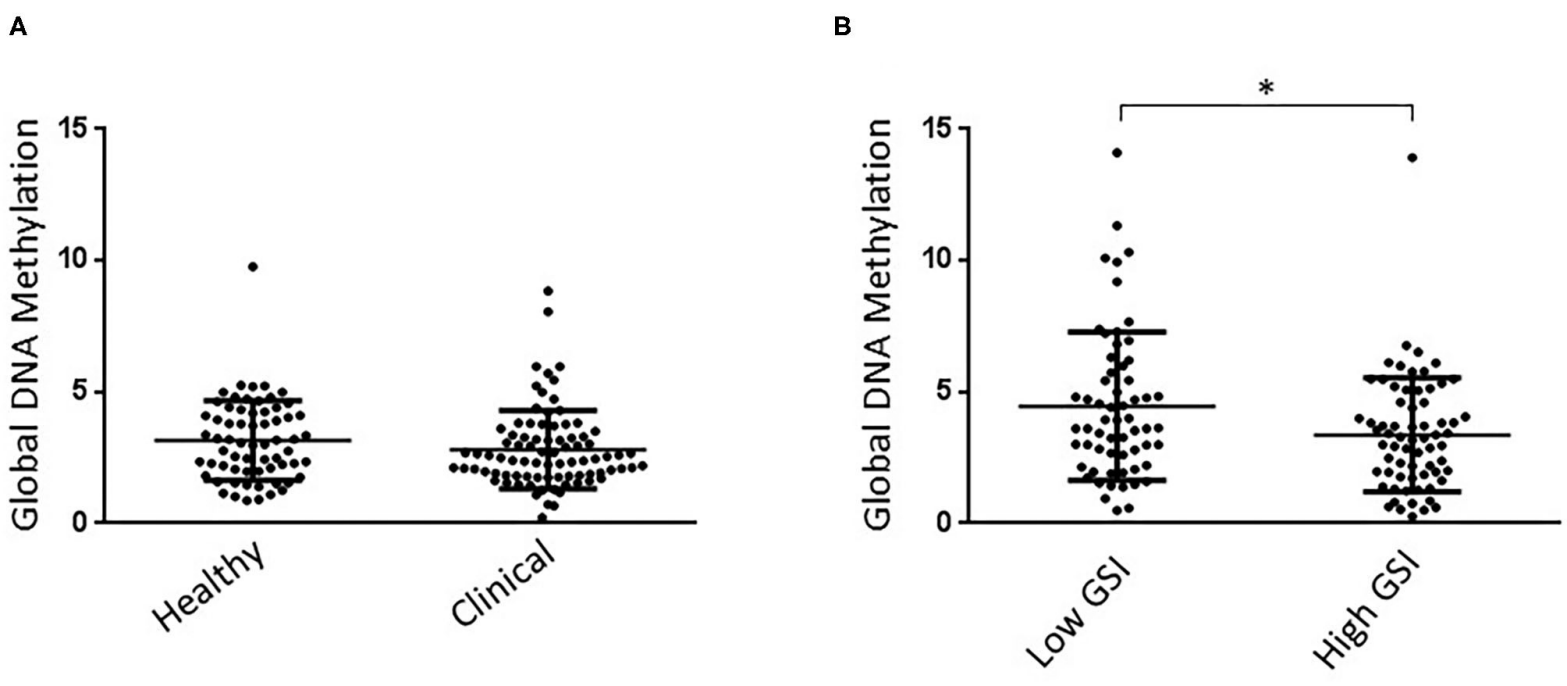
**(A)** Clinical and healthy child groups showed similar Global DNA Methylation levels. No effect of attachment style on global DNA Methylation levels was detected in children. **(B)** High GSI mother groups showed significantly lower Global DNA Methylation levels than low GSI mothers. No effect of child attachment style on global DNA Methylation levels was detected in mothers. *P < 0.05.

Kolmogorov-Smirnov test showed that all measured parameters were normally distributed. ANOVA did not reveal a significant effect of either clinical diagnosis or attachment style, or interaction between these two parameters on global DNA methylation levels. Similar levels of this biomarker were observed between groups [group, *F*_(1, 146)_ =0.010, *p* = 0.921; attachment, *F*_(1, 146)_ = 0.522, *p* = 0.475; group x attachment = *F*_(1, 146)_ =0.771, *p* = 0.382; Figure 1A].

No significant correlations were detected between global DNA methylation and psychological parameters (CBCL/6–18) in children (Supplementary Table 1).

### Global DNA Methylation in Mothers and Its Link to Psychopathological Symptoms

Kolmogorov-Smirnov test showed that all measured parameters were normally distributed. ANOVA showed a statistically significant effect of the GSI group on global DNA methylation levels [group, *F*_(1, 124)_ = 5.664, *p* = 0.019], with high GSI mothers showing lower levels of this biomarker than low GSI mother group (Figure 1B). Further, ANOVA did not reveal a significant effect of either attachment style, or interaction between GSI group and attachment style [attachment, *F*_(1, 124)_ = 0.110, *p* = 0.741; group x attachment = *F*_(1, 124)_ = 0.010, *p* = 0.979].

Significant correlations were observed between this biomarker and the following SCL-90-R subscales: interpersonal sensitivity (*r* = −0.218, *p* = 0.014), hostility (*r* = −0.279, *p* = 0.002), psychoticism (*r* = −0.302, *p* = 0.001, and GSI (*r* = −0.229, *p* = 0.010; Table 1).

**Table 1:** Associations between psychological parameters (SCL-90-R) and Global DNA Methylation in the entire mother sample.

**Maternal SCL90R**	**Global DNA Methylation**	
Somatization	−0.067^#^	0.458^*^
Obsessive-compulsivity	−0.064	0.481
Interpersonal sensitivity	–**0.218**	**0.014**
Depression	−0.145	0.106
Anxiety	−0.016	0.855
Hostility	–**0.279**	**0.002**
Phobic anxiety	−0.012	0.894
Paranoid ideation	−0.025	0.778
Psychoticism	–**0.302**	**0.001**
Global severity index	–**0.229**	**0.010**

# *r, coefficient*.

* *p value; **bold**: significant r coefficient (p < 0.05)*.

### Relation Between Maternal, Children Psychopathological Symptoms, and Global DNA Methylation

Multiple regression analyses showed that maternal GSI score was positively associated with all child symptoms measured by CBCL/6-18. The results of the mediation analyses did not confirm the mediating role of maternal global DNA methylation or child global DNA methylation in the association between GSI and CBCL/6-18. In fact, the direct effects of maternal GSI on child symptoms were still highly significant when controlling for maternal or child global DNA methylation (Supplementary Table 2).

Finally, while the association between maternal and child psychological symptoms was found, the association between maternal and child levels of global DNA methylation was not observed (*r* = −0.045, *p* = 0.655).

## Discussion

The psychobiological mechanisms that mediate childhood psychopathologies are largely unknown. One candidate is DNA methylation, a mechanism that has been frequently associated with psychological and behavioral alterations in adulthood (35), although it has also been implicated in childhood [less frequently; (35, 36)]. Moreover, DNA methylation has been suggested to govern the “translation” of the environmental events and effects on an organism at an early age (10).

Despite this evidence, in our study, we did not observe any alterations in global DNA methylation in clinical children with a diagnosis of DMDD, a rare (2–5%) psychopathological condition that affects children during development. Further, no association between symptom severity and global DNA methylation levels was observed in children. Overall, this result does not support evidence of altered DNA methylation in this pediatric clinical condition. Alternatively, alterations of this biological parameter could be small and specific that could not be detected by dot blot assay. In previous researches, this technique has been successful to detect large changes in global DNA methylation, but it was not sensitive enough to detect more subtle changes (20).

Previously, few clinical studies in children identified changes in the DNA methylation status by both candidate-gene approach and epi-genome-wide technique, showing the existence of such alterations associated with internalizing/externalizing symptoms (7, 37, 38), multiple risk behaviors (39), and attention-deficit hyperactivity disorder-associated symptomatology (40). This result could be explained considering the specific development phase in which we analyzed the children (middle childhood: the children of our sample had 8/9 years). As a matter of fact, this stage of life is very dynamic with many changes occurring in social and emotional processes (7) and that will result into a more stable scenario only in later years. Similarly, DNA methylation is a highly dynamic process at the early stages of development, and it achieves stability only in later stages of maturation (20, 41). Such variability in psychological and biological processes could therefore prevent the observation of a stable relationship between DNA methylation and clinical DMDD symptoms.

In the study conducted in the mother sample, high GSI mothers differed from low GSI mothers in terms of the global DNA methylation levels, the former harboring the lowest levels of this marker. Further, in mothers, this parameter correlated negatively with several psychological symptoms, such as hostility, interpersonal sensitivity, and psychoticism. Overall, this result corroborates previous studies, wherein the levels of DNA methylation were altered in adult psychopathologies such major depression, anxiety disorders, and bipolar disorder (7, 15, 18, 20, 42–44). Specifically, in a recent study, we reported an association between DNA methylation at the dopamine transporter promoter and psychological parameters such as hostility, psychoticism and GSI in adulthood (7).

The absence of an effect of attachment style (mother-child relationship variable) on the DNA methylation in mothers and children is inconsistent with much of the literature, which suggests that this parameter is a mediator of the effects of the early-life environment (e.g., child-mother relationship) on the individual's psychological and behavioral development (20, 45–47). Further, most of these studies reported a more frequent association between DNA methylation and childhood maltreatment and adversity (48)—experiences that can profoundly and dramatically impact a child's development, rather than an association with attachment style. In our study, we did not measure the eventual exposure to childhood maltreatment and thus might have insufficiently proper experimental conditions to detect the effects of early-life experiences on the children and their mothers.

Another relevant aspect of this research is the biological and psychological examination of the mother-child dyad. Interestingly, we found an effect of maternal mental health on the severity of children's symptoms, which was independent of both maternal and child DNA methylation levels. Worst maternal psychopathological status was associated, indeed, to increased severity of symptoms in children. Moreover, despite psychological symptoms were associated between mothers and children, no association was instead found between maternal and offspring's DNA methylation. Overall, these findings do not support the role of this biological biomarker in the transmission of psychological symptoms from mothers to children, or, as already mentioned, its effects were so subtle that they could not be detected by this biological assay.

Previous studies investigating the association between DMDD symptoms and parental psychopathology corroborate our results. These had reported that mothers of DMDD children report mood disorders during their lifetime (49). The co-occurrence between DMDD and maternal depression received robust evidence by several studies (17, 50, 51). It is widely known that maternal and child's emotional/behavioral functioning can be intergenerationally associated in a homotypic (i.e., similar clinical manifestations in mothers and children) and heterotypic manner (i.e., different clinical manifestations between mothers and children) (52). In our sample, mothers and children homotypically shared psychopathological risk especially. Previous studied on families with children with DMDD showed that they experience difficulties in constructing and maintaining positive affective relationships with their caregivers and peers, because these children frequently manifest high irritability and low tolerance to frustration (53). On the other hand, the same studies posited that the mothers of children with DMDD can show poor caregiving capacities. Therefore, we can hypothesize that, consistently with a transactional standpoint, children with DMDD's and their mothers' symptoms could reinforce each other (1).

Our study has several important limitations. First, in this study global DNA methylation was measured by dot blot assay, a method that has several limitations, but also several advantages. This technique does not provide precise information on the methylation status at the single-gene level, compared to epi-genome-wide DNA methylation techniques do. This method determines only large variations in DNA methylation and therefore it is only appropriate for the rough estimation of DNA methylation. However, the dot blot assay is a suitable method to perform a large-scale screening of global DNA methylation in large populations since it requires a small amount of DNA, is less expensive, and allows many samples to be analyzed in parallel (54). These features, together with the ease of implementation, make this assay suitable for the evaluation of this biomarker in clinical contexts. In previous studies, this technique has been successful to detect changes in global DNA methylation in preclinical models and clinical samples (18, 20, 55, 56), but it was not sensitive enough to detect subtle changes (20). In light of these considerations, we cannot rule out that a non-finding, in our study, does not necessarily mean that no differences in methylation distribution across the genome may exist. It is indeed possible that while overall methylation does not change, individual markers may be over or undermethylated.

Another limitation is linked with the use of only the ECR-RC to evaluate the attachment style. Although this tool shows excellent reliability and validity (28) and it is widely used to measure attachment style (57), it may not be sufficient to investigate such a complex construct. Other more structured instruments, such as the Child Attachment Interview (58) and Adult Attachment Interview (59, 60), might be also used to investigate attachment in our mother-child dyads.

Overall, our results describe psychological and biological alterations in the mothers of DMDD children. These findings should encourage the psychological support for mothers of children with DMDD with beneficial effects on their own psychological health and indirectly on the family environment to which the child is exposed every day. To this aim, future studies should assess the efficacy of this therapeutic approach on ameliorating DMDD symptomatology.

In light of the vast clinical and preclinical literature describing a link between maternal behavior, DNA methylation, and child development (61), in future studies, it would be worthwhile to investigate whether these mothers, who show high levels of psychological symptoms and global DNA methylation alterations, provide different levels and/or quality of maternal care to their children. In humans, unlike in preclinical models, maternal care are characterized by many different components, including species-specific behaviors such as nurturing habits (e.g., feeding habits, attachment style, and physical interpersonal touch) as well as cognitive (e.g., meaning-making and appraisal processes) and intersubjective abilities (e.g., sensitivity, responsiveness, and contingent response to infants' signals). Specifically, it would be interesting to characterize the mothers included in this study for specific alterations in these components, and if these changes impact on child development trough DNA methylation.

Finally, although DNA methylation does not appear to be involved in the co-occurrence of psychiatric symptoms in mother and child in the current study, future longitudinal studies would help to examine the role of DNA methylation in the inheritance of vulnerability to depression from mother to child at different stages of the child's development. Eventually, to accurately assess this and cover an important limitation of this study, these future studies should be conducted using an epi-genome wide approach.

## Author Contributions

VC contributed to design the study and to write the manuscript and also analyzed and interpreted the data and prepared the figures. SC designed the study and contributed to write the manuscript and to collect and interpret the data. SB performed the biological assessment and assisted in writing the manuscript. LC contributed to design the study, to write the manuscript, and to interpret the data. RT assisted in designing the study and interpreting the data and also supervised the whole research. All authors have read and approved the manuscript.

## Conflict of Interest

The authors declare that the research was conducted in the absence of any commercial or financial relationships that could be construed as a potential conflict of interest.
