# Supplementary material for: Children With Disruptive Mood Dysregulation Disorder and Psychopathological Risk in Their Mothers: The Function of Global DNA Methylation
Source: Front Psychiatry. 2021 Jan 27;12:593500. doi: 10.3389/fpsyt.2021.593500 (PMC7874238; doi:10.3389/fpsyt.2021.593500)
Supplement: Supplementary file 1 [file Data_Sheet_1.PDF]

Supplementary Table1. Associations between psychological parameters (SCL90R) and Child Global DNA Methylation.

CBCL/6–18	**Global DNA Methylation**	
Anxious/Depressed	-0.105[#]	0.203*
Withdrawn/Depressed	-0.127	0.123
Somatic Complaints	-0.011	0.892
Social Problems	-0.134	0.103
Thought Problems	-0.061	0.463
Attention Problems	-0.108	0.190
Rule-Breaking Behavior	-0.049	0.552
Aggressive Behavior	-0.009	0.914
DSM-Depressive Problems	-0.056	0.498
DSM-Anxiety Problems	-0.042	0.607
DSM-Somatic Problems	-0.050	0.541
DSM-Attention Deficit/Hyperactivity Problems	-0.062	0.455
DSM-Oppositional Deviant Problems	0.044	0.593
DSM-Conduct Problems	-0.112	0.174

r coefficient; * p value
