# Supplementary material for: Children With Disruptive Mood Dysregulation Disorder and Psychopathological Risk in Their Mothers: The Function of Global DNA Methylation
Source: Front Psychiatry. 2021 Jan 27;12:593500. doi: 10.3389/fpsyt.2021.593500 (PMC7874238; doi:10.3389/fpsyt.2021.593500)
Supplement: Supplementary file 2 [file Data_Sheet_2.PDF]

Supplementary Table 2. Multiple regression analyses and mediation analysis of the maternal GSI contribution to child symptomatology, over and above the contribution of maternal and child global DNA methylation

	GSI effect - Multiple Regression-			GSI effect - Mediation Analysis (maternal global DNA methylayion)			GSI effect - Mediation Analysis (child global DNA methylayion)		
CBCL/6-18	Beta	t	p	Beta	t	p	Beta	t	p
Anxious/Depressed	0.65	9.52	< 0.001	0.64	9.13	< 0.001	0.64	9.85	< 0.001
Withdrawn/Depressed	0.59	8.29	< 0.001	0.59	8.04	< 0.001	0.60	8.29	< 0.001
Somatic Complaints	0.68	10.28	< 0.001	0.67	9.98	< 0.001	0.67	10.23	< 0.001
Social Problems	0.69	10.84	< 0.001	0.70	10.73	< 0.001	0.70	10.93	< 0.001
Thought Problems	0.72	11.79	< 0.001	0.72	11.48	< 0.001	0.72	11.77	< 0.001
Attention Problems	0.73	11.88	< 0.001	0.71	11.34	< 0.001	0.73	11.83	< 0.001
Rule-Breaking Behavior	0.70	10.63	< 0.001	0.69	10.35	< 0.001	0.69	10.58	< 0.001
Aggressive Behavior	0.59	8.15	< 0.001	0.58	7.84	< 0.001	0.58	8.12	< 0.001
DSM-Depressive Problems	0.75	12.71	< 0.001	0.74	12.24	< 0.001	0.75	12.68	< 0.001
DSM-Anxiety Problems	0.51	6.74	< 0.001	0.49	6.31	< 0.001	0.51	6.72	< 0.001
DSM-Somatic Problems	0.77	13.59	< 0.001	0.75	13.02	< 0.001	0.77	13.52	< 0.001
DSM-Attention Deficit/Hyperactivity Problems	0.63	9.17	< 0.001	0.61	8.68	< 0.001	0.63	9.12	< 0.001
DSM-Oppositional Deviant Problems	0.47	5.94	< 0.001	0.47	5.90	< 0.001	0.47	5.94	< 0.001
DSM-Conduct Problems	0.72	11.71	< 0.001	0.72	11.31	< 0.001	0.72	11.72	< 0.001
